# Awake Fiberoptic Intubation in a Patient With Esophageal Squamous Cell Carcinoma

**DOI:** 10.7759/cureus.88875

**Published:** 2025-07-28

**Authors:** Ayesha Yousaf, Richard Smilie, Jeremy Fam, John Harman, Satoshi Yamamoto

**Affiliations:** 1 Anesthesiology, University of Texas Medical Branch, Galveston, USA

**Keywords:** awake fiberoptic intubation, awake intubation, esophageal squamous cell carcinoma (scc), precedex, subclavian pseudoaneurysm, surgical case reports

## Abstract

This case report describes the successful use of awake fiberoptic intubation in a 72-year-old female with a history of squamous cell carcinoma (SCC) of the upper esophagus, presenting with hemoptysis secondary to a subclavian pseudoaneurysm. The patient underwent awake intubation using a combination of dexmedetomidine for sedation and topical anesthesia to facilitate intubation while maintaining spontaneous breathing and airway reflexes. This technique proved effective in addressing the patient's complex and precarious medical condition.

## Introduction

Airway management in patients with esophageal or mediastinal tumors poses a significant challenge for anesthesiologists. Bleeding, anatomical distortion, and mass effect are among the complexities that can be further compounded by active hemoptysis and pseudoaneurysms. In such patients, standard approaches to endotracheal intubation may be hindered due to an increased risk of airway collapse, aspiration, and hemodynamic instability [1,2]. As a result, awake fiberoptic intubation (AFOI) provides a pivotal technique in the management of delicate cases, enabling effective airway control while preserving spontaneous ventilation [3].

Esophageal squamous cell carcinoma (ESCC) represents the majority of esophageal cancers worldwide and is commonly associated with aggressive local invasion [4]. Advanced ESCC can result in life-threatening complications such as pseudoaneurysms, fistula formation, and hemoptysis due to nearby vascular invasion [4,5]. A pseudoaneurysm is a contained vascular rupture where blood leaks from the injured site, collects outside the vessel wall, and is confined by surrounding tissues. Due to the fragility of such structures, they are at high risk for rupture and bleeding, especially when adjacent to malignant invasion. Tumor spread may further complicate perioperative airway management, exacerbating the risk of airway collapse, bleeding, or aspiration risk [5].

The American Society of Anesthesiologists and the Difficult Airway Society endorse the use of AFOI in cases where airway obstruction, bleeding, or distortion prevent rapid induction and intubation under general anesthesia [6,7]. Imaging and clinical features become essential in managing the airway in patients with head, neck, or esophageal malignancies that are compounded by tracheal or vascular involvement [8,9].

Awake intubation also helps reduce the risk of aspiration, especially in the context of active hemoptysis [10]. Dexmedetomidine (Precedex), a selective alpha-2 adrenergic agonist, is useful in AFOI procedures because it provides adequate sedation with minimal respiratory depression, thereby preserving airway reflexes and spontaneous ventilation [11-13]. Additionally, fiberoptic guidance allows for real-time visualization of anatomic distortions, enabling safe intubation in cases of tumor invasion or varices [14].

This report highlights the use of dexmedetomidine in AFOI for a 72-year-old female with upper ESCC and a left subclavian pseudoaneurysm presenting with acute hemoptysis. It underscores the success of AFOI in high-risk airway management.

## Case presentation

A 72-year-old female patient presented to the emergency department (ED) with an onset of acute hemoptysis that commenced earlier in the morning. Her medical history was notable for ESCC classified as cT3, cNX, cM0, for which she had undergone treatment with cisplatin and 5-fluorouracil (5FU) chemotherapy. Additionally, she possessed a medical history of diverticulitis, melanoma, and anemia that necessitated near-weekly blood transfusions as a consequence of chemotherapy-related complications. The patient had also undergone the placement of a G-tube by a thoracic surgeon. She reported cessation of smoking approximately 21 years ago, with a prior history of cigarette smoking that initiated around 41 years ago, culminating in a total smoking history equivalent to 20 pack-years. She had never utilized smokeless tobacco products. She indicated current alcohol consumption but denied any illicit drug use. Her familial medical history was significant for cancer in her father, lung cancer in her paternal grandfather, and various cancer-related conditions in her mother.

**Figure 1:**
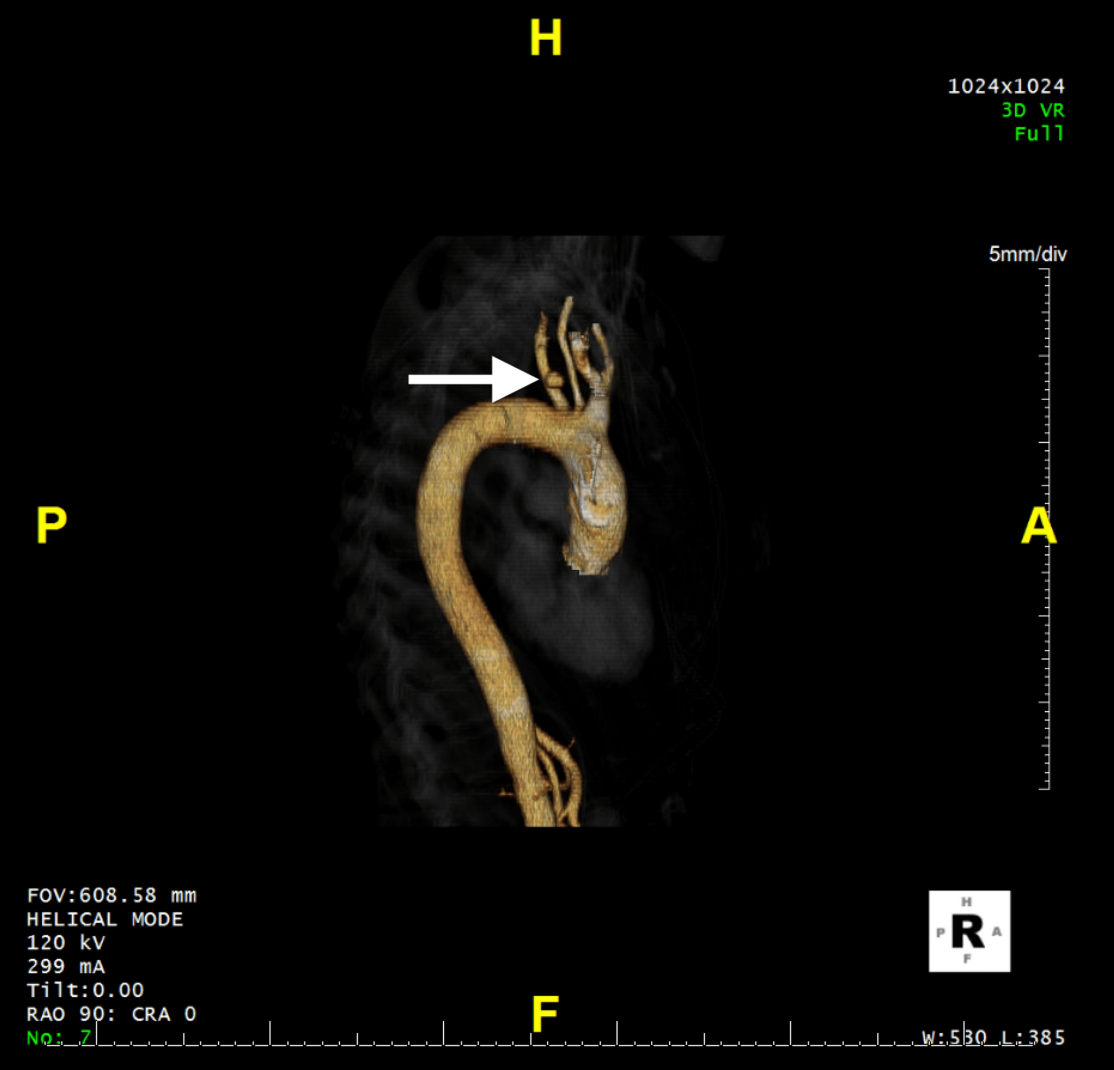
Left subclavian artery pseudoaneurysm visualized on three-dimensional (3-D) CT angiography. 3-D, volume-rendered CT angiogram demonstrating a pseudoaneurysm arising from the proximal left subclavian artery (white arrow), adjacent to the aortic arch.

**Figure 2:**
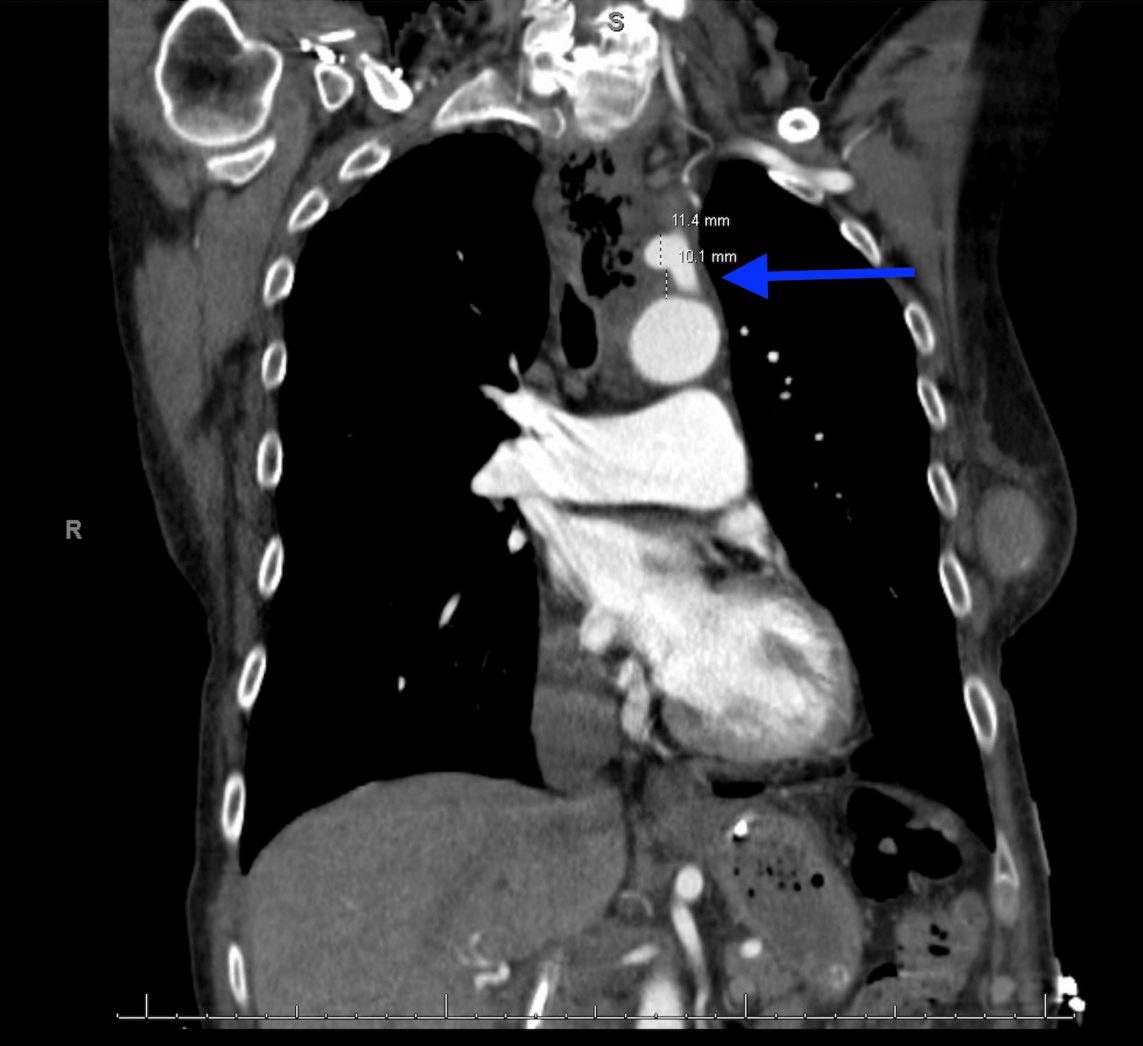
Left subclavian artery pseudoaneurysm due to tumor invasion. Coronal CT image showing a left subclavian artery pseudoaneurysm (blue arrow) measuring 1.2 cm × 1.1 cm × 1.3 cm, located approximately 1.2 cm from its origin off the aortic arch.

Upon her arrival at the ED, the patient exhibited hemodynamic stability but was sporadically expectorating small quantities of blood. Computed tomography (CT) revealed a pseudoaneurysm involving the left subclavian artery and aortic arch, along with progression of the esophageal neoplasm marked by vascular encasement of the left common carotid and subclavian arteries, and obliteration of fat planes at the aortic arch (Figures 1-2). There was noted progressive invasion of the trachea and the presence of pseudoaneurysms originating from the left subclavian artery and the aortic arch, with suspicion of a fistula or extravasation into the esophageal tumor lumen from the left subclavian pseudoaneurysm. Small areas of enhancement within the distal esophagus appeared to be correlated with either bleeding, tumor enhancement, or esophagitis. Additionally, there was an increase in mediastinal adenopathy and the emergence of new small right lung nodules, likely indicative of metastatic disease. Consultation with cardiothoracic surgery was sought in light of these findings. Vascular surgery was also consulted regarding the left subclavian pseudoaneurysm and concerns about an aortic pseudoaneurysm.

The patient reported requiring weekly blood transfusions due to anemia; her hemoglobin level was documented at 9.1 g/dL. She noted that her hemoptysis began on the morning of presentation, initially presenting as the expectoration of mucus that subsequently became bloody. Since her arrival at the ED, she had experienced an escalation in hemoptysis accompanied by persistent coughing. She denied experiencing any chest or arm pain. Her hemodynamic status remained stable, with a blood pressure of 116/68 mmHg, heart rate of 102 beats per minute (bpm), oxygen saturation of 98%, and temperature of 36.7 °C (98 °F). A full summary of the patient's labs can be found in Table 1. The patient was dependent on a GJ tube and did not ingest anything orally. Surgical intervention was scheduled for the same day, involving thoracic endovascular aortic repair (TEVAR) and stenting of the left subclavian artery, as well as addressing a possible descending aortic pseudoaneurysm, which may be attributable to radiation effects or tumor erosion.

**Table 1:** Complete blood count (CBC) and coagulation panel results. WBC, white blood cell count (×10³/µL); RBC, red blood cell count (×10⁶/µL) (low); HGB, hemoglobin (g/dL) (low); HCT, hematocrit (%) (low); MCV, mean corpuscular volume (fL); MCH, mean corpuscular hemoglobin (pg); MCHC, mean corpuscular hemoglobin concentration (g/dL); RDW-SD, red cell distribution width-standard deviation (fL) (high); RDW-CV, red cell distribution width-coefficient of variation (%) (high); PLT, platelet count (×10³/µL) (low); MPV, mean platelet volume (fL); IPF, immature platelet fraction (%); PT, prothrombin time (seconds); PT-INR, prothrombin time-international normalized ratio

Lab	Results	Reference ranges
CBC		
WBC ×10^3^/µL	7.03	4.3-11
RBC ×10^6^/µL	3.06	3.9-5.25
HGB (g/dL)	9.1	11.6-15
HCT (%)	26.6	35.7-45.2
MCV (fL)	86.9	80.6-95.5
MCH (pg)	29.7	25.8-32.8
MCHC (g/dL)	34.2	31.6-35.1
RDW-SD (fL)	52.3	39-49.9
RDW-CV (%)	16.6	12-15.5
PLT ×10^3^/µL	108	166-358
MPV (fL)	10	9.5-12.9
IPF (%)	2.4	1.3-7.7
Coagulation		
PT (s)	11.4	10.1-12.6
PT-INR	1	<1.1

**Figure 3:**
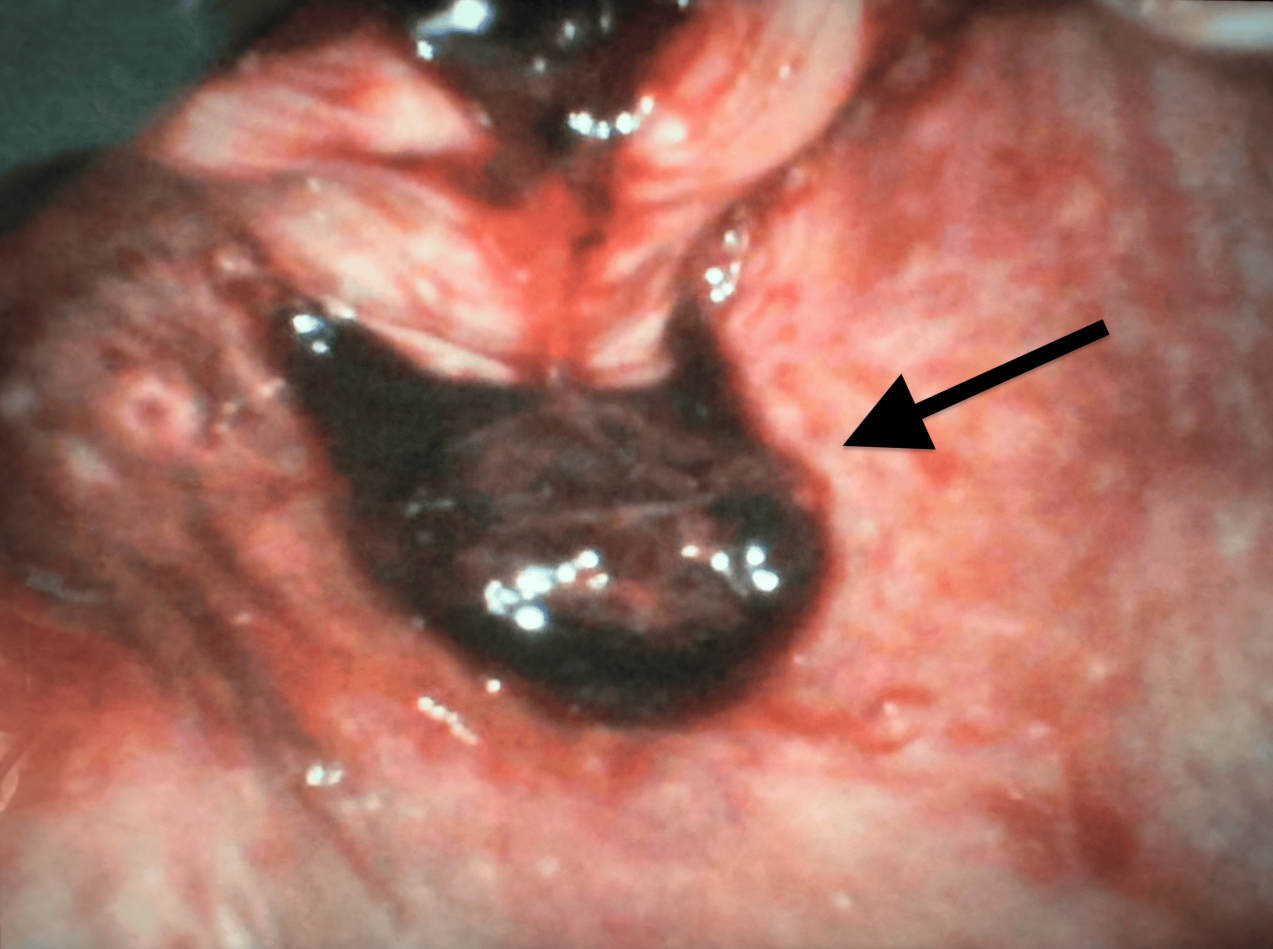
Prominent esophageal varices secondary to superior vena cava (SVC) obstruction. Endoscopic image demonstrating prominent mucosal varices (black arrow) in the upper esophagus. In this case, the varices are attributed to obstruction of the SVC due to esophageal squamous cell carcinoma (ESCC) invasion into the mediastinum, resulting in impaired venous drainage and development of *downhill* varices.

In the operating room (OR), AFOI was selected for this patient to preserve airway reflexes and spontaneous respiration while ensuring adequate sedation and topical anesthesia. The Glidescope system was prepared and readily available as a backup but was ultimately not needed. The procedural steps included:

(1) Administration of pre-sedation with dexmedetomidine (Precedex), with a loading dose of 0.5 mcg/kg over 10 minutes, followed by a maintenance infusion of 0.5 mcg/kg/hour.

(2) Topical anesthesia of the vocal cords and subglottic region through two applications of local anesthetic sprays. The patient gargled the solution to adequately anesthetize the posterior pharyngeal wall.

(3) Insertion of a 7.0 endotracheal tube (ETT) utilizing the Glidescope fiberoptic system.

(4) Confirmation of ETT placement at 24 cm at the lips.

(5) Slight migration of the ETT during withdrawal of the fiberoptic scope, followed by adjustment and confirmation of correct placement using the Glidescope MAC S3 under direct visualization.

(6) Identification of significant varices on the posterior wall of the esophagus, adjacent to the epiglottis (Figure 3).

The awake intubation technique was proficiently performed, with the patient exhibiting comfort and cooperation during the entirety of the procedure. This methodology significantly reduced the potential hazards linked to general anesthesia in a patient experiencing considerable hemorrhage and impaired airway integrity. The administration of dexmedetomidine yielded sufficient sedation without compromising respiratory function, while local topical anesthesia ensured optimal analgesia of the airway anatomical structures. Continuous hemodynamic monitoring was maintained via a pre-induction arterial line, ensuring real-time assessment of stability throughout the intubation process.

## Discussion

This case exemplifies the critical significance of awake intubation in intricate clinical scenarios where the preservation of spontaneous respiration and airway reflexes is paramount. The favorable outcome observed in the patient accentuates the effectiveness and safety of this method in addressing airway complications associated with considerable hemoptysis and pseudoaneurysms [1,3].

AFOI presents with numerous advantages in comparison to conventional intubation techniques, particularly in patients with compromised airways or potential bleeding. Primarily, it facilitates continuous visualization of the airway, which is essential for the accurate identification and navigation around obstructions or anatomical anomalies [14]. In the context of this patient's case, the detection of substantial varices on the posterior wall of the esophagus was pivotal in directing the intubation procedure.

Given the unknown source and extent of the patient's bleeding, there was significant concern regarding the potential for airway collapse and the inability to secure the airway following induction of general anesthesia. In order to mitigate the risk of encountering a cannot-intubate/cannot-ventilate scenario, AFOI was selected as the safest approach. AFOI significantly reduces the risk of aspiration, a consideration of utmost importance in patients experiencing active bleeding or excessive secretions [10]. By enabling the patient to maintain spontaneous breathing and protective airway reflexes, the likelihood of aspiration during the procedure is markedly diminished [7].

The incorporation of dexmedetomidine in AFOI provides the added benefit of delivering sedation without compromising respiratory function. Dexmedetomidine is recognized for its capacity to induce sedation while safeguarding airway reflexes and spontaneous respiration, rendering it an optimal selection for patients requiring intubation who are susceptible to respiratory depression from alternative sedative agents [11-13]. Dexmedetomidine's potential side effects, including bradycardia and hypotension, did not preclude its use in this case, as continuous hemodynamic monitoring via an arterial line ensured patient stability throughout the procedure [13]. 

The successful resolution of this intricate intubation further emphasizes the need for meticulous preparation and technical expertise when performing AFOI. The administration of sufficient topical anesthesia and the utilization of appropriate instruments, such as the Glidescope fiberoptic and MACS3, were vital in ensuring patient comfort and cooperation [15]. The immediate reintubation following endotracheal tube dislodgment also underscores the importance of vigilance and preparedness in swiftly addressing potential complications.

## Conclusions

AFOI represents a valuable technique in the management of complex airway cases, particularly among patients experiencing intermittent airway bleeding or anatomical challenges. The synergistic use of dexmedetomidine and topical anesthesia offers an effective and safe strategy for maintaining airway patency while mitigating the risks associated with general anesthesia. This case illustrates the successful implementation of awake intubation in a patient with ESCC and sporadic hemoptysis, thereby demonstrating its efficacy in navigating airway challenges within the critical care environment.
